# Lack of c-kit receptor promotes mammary tumors in N-nitrosomethylurea-treated Ws/Ws rats

**DOI:** 10.1186/1475-2867-8-5

**Published:** 2008-04-29

**Authors:** Maricel V Maffini, Ana M Soto, Carlos Sonnenschein, Nikoletta Papadopoulos, Theoharis C Theoharides

**Affiliations:** 1 Department of Anatomy and Cellular Biology, Tufts University School of Medicine, Boston, USA; 2 Department of Pharmacology and Experimental Therapeutics, Biochemistry and Internal Medicine, Tufts University School of Medicine, and Tufts-New England Medical Center, Boston, USA

## Abstract

**Background:**

c-*kit *is expressed in various cell types during development and it has been linked to the promotion of cellular migration, proliferation and/or survival of melanoblasts, hematopoietic progenitors and primordial germ cells. Several reports have proposed a role for the c-*kit *gene on carcinogenesis. Gain-of-function mutations are associated with diseases such as mastocytosis and gastrointestinal stromal tumors among others. However, very little is known about pathologies associated with loss-of-function mutations. Regarding breast cancer, c-kit protein and mRNA are highly expressed in normal breast but their expression decreases or is absent in the presence of breast cancer. We studied the role of *c-kit *in mammary carcinogenesis in the Ws/Ws rats carrying spontaneous lack-of-function mutation in the c-*kit *gene. Fifty day-old virgin female Ws/Ws rats and their wild type counterparts were injected with either 50 mg/kg body weight of the chemical carcinogen N-nitrosomethylurea or with vehicle. The animals were followed-up for 6 months. Fisher 344 rats were used as positive controls for tumor development.

**Results:**

Eleven weeks after treatment, palpable tumors were detected in the Ws/Ws rats. The tumor incidence was 80% in Ws/Ws rats, while no tumors were observed in the wild type rats (p = 0.006). Our data show that the lack of c-kit is permissive for the development of mammary tumor in Ws/Ws rats treated with carcinogen.

**Conclusion:**

We conclude that the lack of c-kit may contribute to an imbalanced homeostatic state in the mammary gland either by affecting signaling between stroma and epithelium, or through the lack of mast cells.

## Background

Several reports have proposed a role for the c-*kit *gene on carcinogenesis. The c-*kit *gene encodes a protein that is a member of the tyrosine kinase receptor family. One known ligand for this receptor is the stem cell factor (SCF) [1]. c-*kit *is expressed in various cell types during development, namely, endothelial, epithelial and endocrine cells and it has been linked to the promotion of cellular migration, proliferation and/or survival of melanoblasts, hematopoietic progenitors and primordial germ cells [2].

Spontaneous c-*kit *mutations have been described in mice [3], rats [4] and humans [5]. In rodents, c-*kit *is encoded by the W locus where several mutant alleles have been reported. Rats carrying deletions in both alleles of the c-*kit *gene at the W locus are called Ws/Ws (for white spotting); they have black eyes and white coats [4]. In addition to the depletion of skin melanocytes, the Ws/Ws rats show macrocytic anemia and depletion of mast cells and interstitial cells of Cajal [6]. Contrary to the W mutant mice, no germ cell depletion has been observed in these rats allowing them to be fertile [7].

Both activating and inactivating mutations of c-*kit *have been described. Gain-of-function mutations are associated with mastocytosis and gastrointestinal stromal tumors among other diseases [6,8,9]. However, very little is known about pathologies associated with loss-of-function mutations. Kitamura et al. [6] observed spontaneous forestomach papillomas and antral ulcers in W/W^v ^mice carrying c-*kit *loss-of-function mutation. These lesions were attributed to spontaneous bile reflux due to the depletion of interstitial cells of Cajal that regulate the rhythmical contraction of the gastrointestinal tract, reinforcing the notion that pathologic lesions may arise as a consequence of a disruption in the normal cell interactions of the stomach wall.

Mast cells are believed to play a role in the maintenance of epithelial and connective tissue integrity. Under physiological conditions, mast cells can generate and release numerous potent mediators (i.e. heparin, proteases, cytokines, etc), thus directly or indirectly affecting tissue growth, differentiation and survival. Some of these functions appear to occur via the interaction between the tyrosine kinase c-kit receptor present in the mast cells and its ligand SCF produced by cells such as endothelial cells, fibroblasts, melanocytes, etc. [10].

Mast cells may also be important players during tissue remodeling. For instance, bone density and microstructure of mast cell deficient mice are comparable to those of their wild type counterpart. However, mast cell deficient mice showed marked delay in onset and decreased duration of remodeling, as well as diminished synthesis of new bone matrix [10]. During the physiological remodeling of the mammary gland that occurs during pregnancy and lactation, an increased number of mast cells were observed at the time when the mammary stroma is replaced by lobulo-alveolar structures; however, the number of mast cells declines dramatically during lactation when the gland is occupied exclusively by lactiferous units [11].

As mentioned above, Ws/Ws rats are depleted of mast cells as a consequence of lacking c-*kit*. In order to further clarify any association between mast cells, lack of c-*kit *expression and cancer, we studied mammary tumorigenesis in the context of c-kit null environment in the Ws/Ws rats and their wild-type counterparts using a classical model of chemical carcinogenesis induced by N-nitrosomethylurea (NMU).

## Results

### Ws/Ws and wild type mammary gland morphology

**Figure 1:**
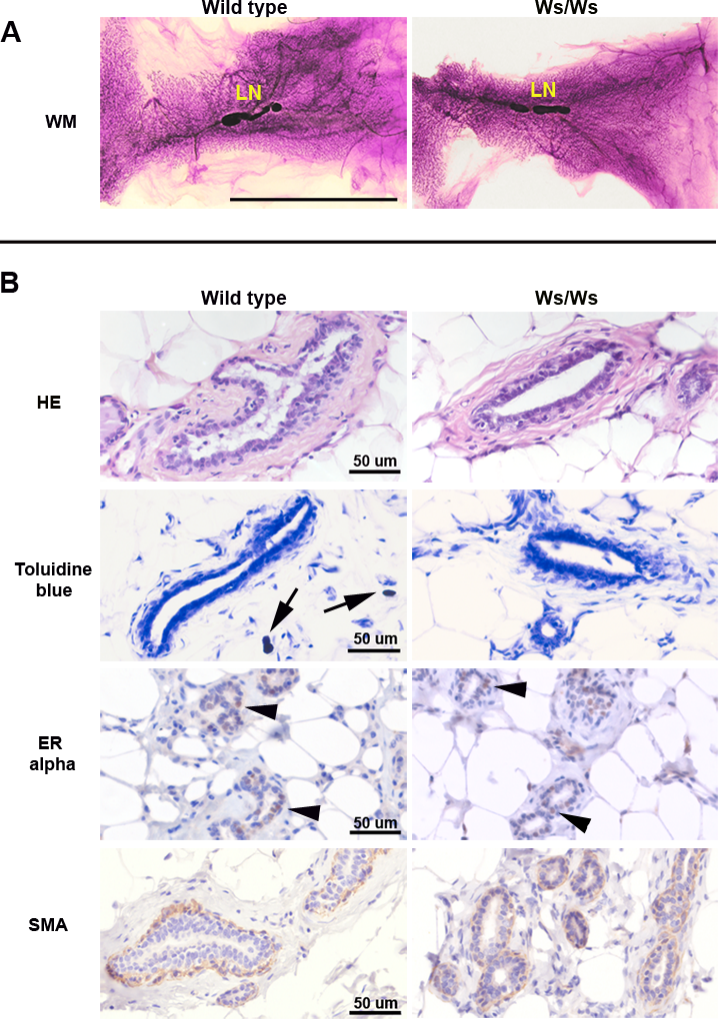
Characterization of Ws/Ws and wild type adult rat mammary glands. Panel A: Mammary whole mounts of normal wild type and Ws/Ws rats. Both glands show a dense ductal network with multiple branching points. Panel B: Histological sections of the glands depicted in Panel A. As expected, the only morphological difference is the absence of mast cell in the Ws/Ws animals. LN: lymph nodes. Ws/Ws: c-*kit *null rats. Immunohistochemistry: positive cells stained brown; Harris's hematoxylin was used as counterstaining. Scale bars: Panel A: 3.2 cm; Panel B: 50 μm.

Whole mounts of abdominal/inguinal mammary glands obtained from approximately 8 month-old animals (rats were euthanized at the end of the experiment, 6 months after carcinogen or vehicle exposure) showed that both Ws/Ws and wild type glands have a dense ductal tree with multiple side-branching and alveolar buds (Figure 1A). Histologically, the mammary glands from both Ws/Ws and wild type rats were indistinguishable; also, both expressed estrogen receptor alpha and had a continuous layer of myoepithelial cells surrounding the luminal epithelial cells (Figure 1B). The only histological difference observed was the lack of mast cells in the Ws/Ws rats (Figure 1B).

### Tumor latency and incidence

None of the wild type rats treated with NMU developed palpable tumors throughout the duration of the experiment. In NMU-treated Ws/Ws rats the first tumor was palpated 11 weeks after the carcinogen injection. Tumors in the positive control F344 rats were palpable starting 10 weeks after treatment (Table 1).

**Table 1:** Tumor incidence in Ws/Ws and wild type rats. Fisher 344 rats were used as a positive control.

Rat #	Tumors removed	Latency (weeks)	Microscopic neoplastic lesions
**Ws/Ws**			
1	YES	19	YES
2	YES	19	YES
3	NO	-	NO
4	YES	12	YES
5	YES	11	YES
**Wild type**			
1	NO	-	NO
2	NO	-	NO
3	NO	-	NO
4	NO	-	NO
5	NO	-	NO
6	NO	-	NO
**F344**			
1	YES	10	YES
2	NO	-	YES
3	NO	10	YES
4	YES	10	YES

**Figure 2:**
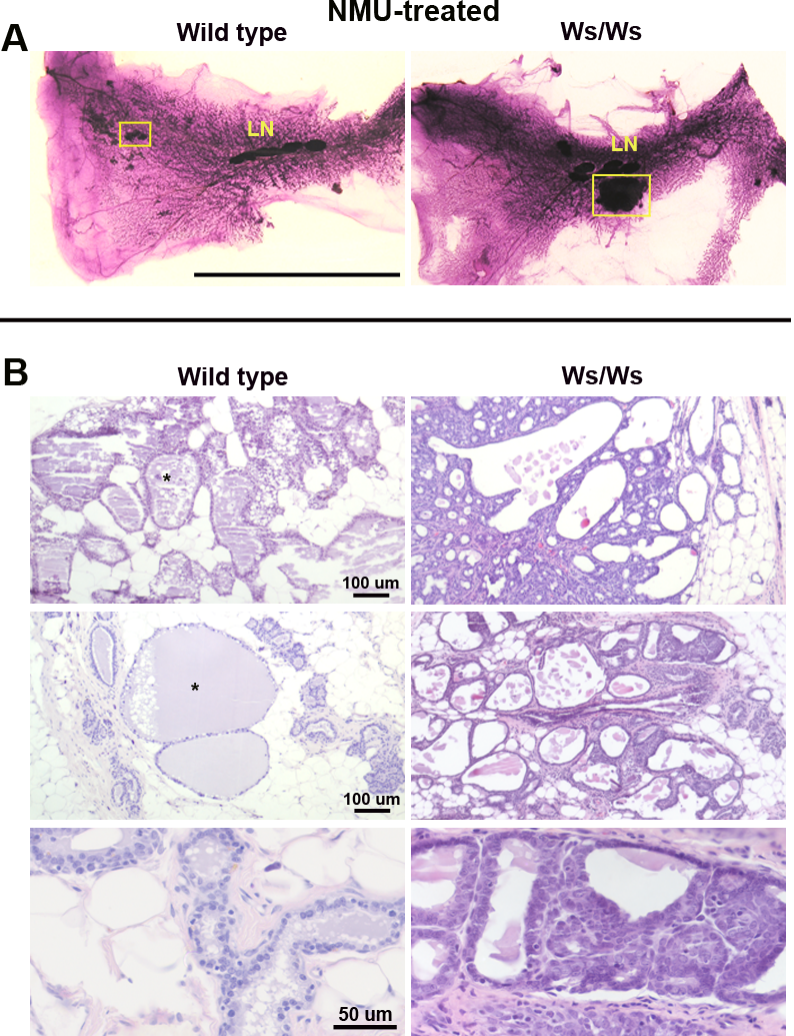
Carcinogen-treated Ws/Ws rats developed mammary carcinomas. Panel A: whole mounts of mammary glands obtained from NMU-treated wild type and Ws/Ws rats. Only the mutant rats developed tumors while the microscopic lesions observed in the wild type mammary glands were benign lesions. Panel B: Histological sections of the region of interest outlined (yellow lines) in the whole mounts. The wild type rats showed large dilated ducts containing secretion (asterisks) whereas the Ws/Ws developed frank carcinomas. Sections were stained with hematoxylin and eosin. LN: lymph nodes. Ws/Ws: c-*kit *null rats. Scale bars: Panel A: 3.2 cm; Panel B: 100 μm and 50 μm.

The total incidence of tumors, including palpable and microscopic ones, in Ws/Ws rats was 80%. There was no statistically significant difference with the positive control F344 rats (p = 0.343). Tumors were palpable in 4 of the 5 Ws/Ws rats and up to three tumors per rat were recorded. In addition to the palpable tumors, the mammary gland whole mounts of the Ws/Ws rats showed numerous microscopic lesions (Figure 2A). These lesions were removed, paraffin embedded and analyzed; the histopathological evaluation confirmed their neoplastic nature (Figure 2B Ws/Ws).

The whole mounts from the NMU-treated wild type mammary glands also showed microscopic lesions (Figure 2A). However, upon histopathological examination those lesions were classified as not neoplastic (Figure 2B wild type). These results are statistically different from the Ws/Ws (p = 0.006) and F344 (p = 0.002) tumor incidence.

None of the vehicle-treated animals, either Ws/Ws or wild type, showed neoplastic lesions during the duration of the experiment. Necropsy was performed in all NMU-treated animals at the end of the experimental observation; no macroscopic tumors were observed in organs other than the mammary glands.

### Mammary gland histopathology

Tumors and microscopic lesions were classified following the criteria described by Russo et al [12]. The tumors observed in Ws/Ws rats were described as adenocarcinoma and carcinoma of cribriform type, ductal carcinoma and fibroadenoma. Figure 2B (Ws/Ws) are representative sections of these tumors. One of the carcinomas had both lobular and ductal components. The mammary glands of the rat that did not develop neoplasia presented dilated and hyperplastic ducts. Tumors developed in the F344 positive control animals were classified as carcinomas of papillary or cribriform type, the most commonly observed tumor types in NMU-treated rats [12].

The mammary glands of NMU-treated wild type rats showed no signs of neoplasia. However, three mammary glands of these nulliparous rats had ductal secretion (Figure 2B, wild type), one also showed dilated and hyperplastic alveolar structures with abundant secretion resembling milk (Figure 2B wild type). The dilated ducts were similar in both Ws/Ws and wild type mammary glands. None of the wild-type rats developed preneoplastic lesions such as intraductal hyperplasias and did not show differences in mast cell numbers compared to vehicle controls (data not shown).

### c-kit expression

**Figure 3:**
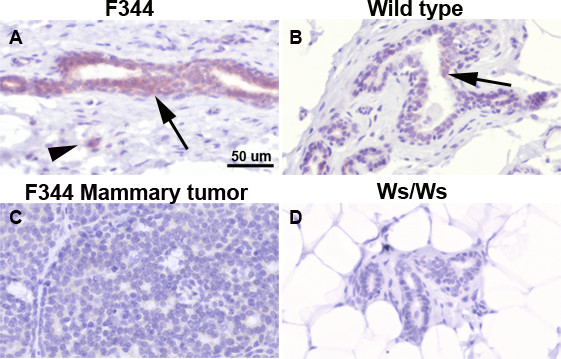
Immunostaining for c-kit. Panels A and B: c-kit is expressed in the cytoplasm of luminal epithelial cells (arrows) and mast cells (arrowhead) present in the stroma of control F344 and wild type rat mammary glands. Panel C: c-kit is not expressed in a mammary tumor obtained from a control F344 rat. Panel D: as expected, Ws/Ws rats do not express c-kit. Counterstaining: Harris' hematoxylin. Ws/Ws: c-*kit *null rats. Scale bar: 50 μm

Immunostaining for c-kit (CD117) was performed in normal mammary glands from Ws/Ws and wild-type rats. Normal mammary glands and mammary tumors from F344 rats were solely used as controls for the quality of NMU preparation and immunostaining. Luminal epithelial cells from F344 rats showed the highest cytoplasmic and membrane c-kit expression, followed by those in the wild type rats (Figure 3A and 3B). As expected, the Ws/Ws mammary glands did not stain for c-kit (Figure 3D). In addition, the mammary tumors from F344 control rats revealed a complete lack of c-kit expression (Figure 3C) which is in agreement with reports describing the loss of c-kit in breast cancer [13].

## Discussion

Our data show that the lack of c-kit is permissive for the development of mammary tumor in Ws/Ws rats treated with the carcinogen NMU. Moreover, wild-type rats, in which c-*kit *is present, did not show signs of neoplasia. Although the number of animals used in this experiment seems small, the observed differences are statistically significant, biologically relevant and plausible. In this regard, NMU is known to induce neoplasia in susceptible strains at the dose used (as it is demonstrated by the tumors developed in our positive control F344 rats) and not to induce mammary tumors in resistant strains (i.e. Copenhagen, Wistar-Kyoto). Hence, this is a reliable model chosen precisely for the high tumor incidence observed in the susceptible strains and absence of effect in the resistant ones, thus allowing comparisons using a small number of animals per group.

The rationale for using Ws/Ws rats instead of the W/W^v ^c-*kit *null mice to study mammary gland carcinogenesis derives from the fact that c-*kit *mutant mice lack functional ovaries [3], and thus are unlikely to develop an estrogen-dependent tumor. Moreover, the rat model is considered overall to be the most suitable animal model due to the many similarities to the human disease regarding hormone-dependence and histopathology [14,15].

A link between c-*kit *gain of function mutations and neoplasms such as mast cell tumors, gastrointestinal stromal tumors, seminoma/dysgerminoma and small cell lung carcinoma has been reported [6,8,16,17]. However, to our knowledge, other than the spontaneous gastric papillomas, no stringent correlations between c-*kit *loss of function mutations and spontaneous tumors have been found. Herein, we observed for the first time that c-*kit *null rats develop mammary carcinomas after treatment with NMU, a chemical commonly used in mammary carcinogenesis studies.

Among the possible explanations for tumor development in c-*kit *null rats, there are two that deserve particular attention: 1) the lack of a "protective" effect due to the absence of the c-kit tyrosine kinase receptor which may contribute to a disruption in the cell-cell communication between c-kit expressing mammary epithelial cells and SCF-expressing fibroblasts, or mast cells and fibroblasts, and 2). the lack of a "protective" effect due to the absence of mast cells. Because these rats are outbred, we cannot yet rule out the effect of mast cells in carcinogenesis given that a mast cell or bone marrow transplantation from wild type to c-*kit *null rats is unfeasible. A way to overcome this shortcoming would be to test this hypothesis in a three-dimension tissue culture setting where mast cells, epithelial cells and fibroblasts are co-cultured.

It is plausible that the lack of c-kit receptor may have additional covert phenotypic effects in addition to the already described anomalies, including the absence of mast cells. c-kit is expressed on the membrane and/or cytoplasm of ductal and alveolar epithelial cells in normal breast [18]. Protein and mRNA expression of c-kit is high in normal breast, significantly lower in carcinomas *in situ *and almost completely undetectable in invasive breast cancer [13]. Benign breast lesions also showed a decreased expression of c-kit but of lower magnitude than that observed in malignant lesions [19-21]. Thus, it has been suggested that the process of malignant progression correlates with the progressive loss of c-kit and that this may reflect the role played by the c-kit/SCF pathway in the maintenance of normal growth of mammary epithelium [13,20]. In this regard, Samoszuk and Corwin showed that when imatinib mesylate (Gleevec) was used to inhibit tyrosine kinase activity, including c-kit, there was an acceleration in the growth rate of mammary adenocarcinomas in mice while the number of mast cells was similar in both treated and untreated animals [22]. A similar association between decrease expression of c-kit and tumor incidence was observed in the thyroid. While low levels of c-kit are expressed in normal thyroid glands and in 60% of benign lesions, the receptor remained undetectable in 60–90% of the follicular and papillary carcinomas [23].

Mast cells, together with other innate immune cells, have been associated with cancer development by mechanisms that include induction of DNA damage by free radicals, paracrine regulation of intracellular pathways, promotion of angiogenesis and tissue remodeling, etc. [24]. However, mast cells have also been described as the "Jekyll and Hyde" of tumor growth, suggesting that they may have different roles in different environments [25]. Regarding patients with breast cancer, several publications have associated mast cells with both poor and good prognosis. Kashiwase et al. suggested that mast cells may play a deleterious role in breast cancer because a significantly greater number of them were found in malignant lesions compared to benign lesions [26]. In contrast, Aaltomaa et al. [27] reported that a high number of mast cells in the tumor stroma significantly correlated with a favorable prognosis. More recently, mast cells in the peritumoral stroma of human breast cancer were correlated with good prognosis [28].

Tanooka et al. showed that after subcutaneous injection of methylcholanthrene, the primary subcutaneous tumor incidence was significantly higher in mast cell-deficient mice than in their wild type counterparts; additionally, after bone marrow transplantation to replenish mast cells, the tumor incidence decreased to that observed in the wild type mice [29], concluding that the higher tumor incidence in the mutant mice was due to the lack of mast cells. The authors also observed that the growth rate of these tumors was similar in both types of mice, suggesting that the putative protective effect of mast cells would become null after the tumors grew to a certain size [29]. Our results using the c-*kit *null rats are in general agreement with this report. The main difference between these two experiments is that none of the wild type rats in our study developed neoplasias.

A close relationship between mast cells and their surrounding stroma has been reported. For instance, heparin from mast cells affects the conversion of soluble collagen to collagen fibers in vitro [30]. Also, mast cells are in close contact or attached to fibroblasts and they enhance fibroblast-mediated contraction of collagen via direct cell-cell contact due in part by the c-kit/SCF interactions [31]. Depletion of mast cells also reduced proteases and protease-activators involved in tissue remodeling [32]. Finally, the role played by epithelial c-kit receptor and its SCF ligand produced by fibroblasts in stroma-epithelium communication in the mammary gland remains unknown.

Altogether, our data suggest that 1) the absence of c-kit receptor in the epithelial cells and 2) the lack of mast cells would affect the homeostatic stroma-epithelial relationships. Therefore, proper stroma remodeling and epithelium maintenance would not occur, thus contributing to the high tumor incidence observed in c-*kit *null rats. One might speculate that the mammary tumor susceptibility observed in these rats may be due to altered tissue interactions that are only manifested when the animals are challenged with the carcinogen NMU [33,34]. Under similar conditions, the wild type rats seem to be able to overcome the carcinogenic effect of NMU. To our knowledge, both Brown Norway and Donryu rats develop tumors [35]. Thus we hypothesize that the "tumor resistance" observed in these wild type rats may be due to the presence of an intact interaction between the different components of the mammary gland. These important yet to be uncovered mechanisms are the object of our current research.

## Conclusion

In summary, mast cell depletion is a consequence of lack of c-*kit*. This receptor has a broad physiological function mediated in part by the interaction with its ligand SCF. The lack of c-*kit *may contribute to an imbalanced homeostatic state in organs that undergo extensive remodeling either directly, by affecting signaling between stroma and epithelium, and/or indirectly, by the lack of mast cells.

## Methods

### Animals

The wild type rats are of the Brown Norway and Donryu genetic background, from which mutant Ws/Ws rats were obtained. The generation of Ws/Ws rats was described elsewhere [4]. Wild type and Ws/Ws rats are outbred and they were purchased from Japan SLC, Inc (Hamamatsu, Shiziuoka Prefecture, Japan). Fisher 344 (F344) rats were purchased from Harlan (Indianapolis, IN). The animals had access to food and water *ad libitum *and were maintained on a 14:10 hours light:dark cycle. Care and procedures were in accordance with the Guidelines for the Care and Use of Animals and the Tufts-New England Medical Center Institutional Animal Care and Use Committee.

### Carcinogen treatment

A single dose of 50 mg/kg body weight of the direct acting carcinogen NMU (Sigma, St. Louis, MO) was injected intraperitoneally to virgin 52 day-old female Ws/Ws (n = 6) and wild-type rats (n = 6). A similar number of animals were injected with vehicle (0.85 g/l sodium chloride, pH 5) as negative controls. To our knowledge, there is no data indicating how Ws/Ws or their wild type counterpart will respond upon exposure to the carcinogen NMU. Therefore, tumor-susceptible F344 rats were injected with the same dose of NMU and served as positive control for the effect of NMU on mammary carcinogenesis.

Starting 1 month after treatment, the animals were palpated weekly and the tumor latency period was recorded. Tumors were removed when they reached 1 cm in diameter. If they reached this size before the termination of the experiment (6 months after carcinogen injection) the tumor was removed and those animals were kept alive until the end of the experiment. The animals were sacrificed and thoracic and abdominal/inguinal mammary glands were removed. One Ws/Ws animal died shortly after the NMU injection, probably as a result of toxicity by the carcinogen. No necropsy could be performed. Consequently, the final number of Ws/Ws rats was 5.

### Tissue processing and stainings

Abdominal/inguinal mammary glands were whole-mounted, fixed in phosphate-buffered formalin and stained with carmine alum as described before [34]. In addition, thoracic mammary glands and tumors were fixed in phosphate-buffered formalin and paraffin embedded. Five microns thickness tissue sections were stained with hematoxylin and eosin or toluidine blue (pH 2) to evaluate the presence of mast cells. The samples were blindly analyzed by a pathologist.

### Immunohistochemistry for c-kit

An antigen retrieval method using microwave pretreatment and 0.01 M sodium citrate buffer (pH 6) was performed as previously described [36]. A polyclonal rabbit antibody for c-kit (CD 117, Dako, Carpinteria, CA) was used at 1:150 dilution. The antigen-antibody reaction was visualized using the streptavidin-peroxidase complex, with diaminobenzidine tetrahydrochloride (Sigma-Aldrich) as the chromogen. Counterstaining was performed with Harris' hematoxylin and the sections were analyzed using an Axioskop 2 Plus microscope (Carl Zeiss, Munchen-Hallbergmoos, Germany) and an AxioCam HR color digital camera (Carl Zeiss).

#### Statistics

Chi-square analysis was performed to compare tumor incidence between positive control F344 rats and Ws/Ws or wild type rats. The same test was used to compare incidence between Ws/Ws and wild type animals.

## Competing interests

The authors declare that they have no competing interests.

## Authors' contributions

MVM, AMS, CS and TCT contributed in the experimental design. MVM and NP carried out the animal work, histology and sample staining. MVM analyzed the data and wrote the manuscript. All the authors read and discussed the manuscript.
